# Metastasis of Uterine Leiomyosarcoma to the Breast: Medical and Histopathological Criteria

**DOI:** 10.1155/2020/8037646

**Published:** 2020-12-18

**Authors:** Eugenia Colón

**Affiliations:** ^1^Department of Pathology, Unilabs, St. Görans Hospital, Stockholm, Sweden; ^2^Department of Women's and Children's Health, Karolinska Institutet, Solna, Sweden

## Abstract

It is uncommon for extramammary tumors to metastasize to the breast, and very few cases describing metastasis of primary uterine leiomyosarcoma to the breast have been reported. We present the case of a 51-year-old woman diagnosed with metastasis of uterine leiomyosarcoma to the breast diagnosed 10 years ago after hysterectomy. Ultrasonography, mammography, and cytology were used to establish a preliminary diagnosis that was confirmed upon examination of the excised tumor that show a rare soft tissue tumor composed of atypical spindle cells and increased proliferation rate. We discuss the importance of distinguishing between various primary mesenchymal tumors of the breast because of phenotypic overlap and some guidance of the histological criteria for metastasis of leiomyosarcoma, as well as differential diagnosis and surgical treatment.

## 1. Introduction

Breast metastases from extramammary malignancies are uncommon, representing approximately 2% of all breast tumors [1–4], perhaps due to the fibrous nature and low vascularity of the breast [4–7]. Uterine leiomyosarcoma is a rare mesenchymal neoplasm accounting for 1.3% of all uterine malignancies and 30% to 40% of all uterine sarcomas [1].This report adds to a handful of reports describing the metastasis of uterine leiomyosarcoma to the breast.

In the case under study in this report, the patient had undergone a salpingooophorectomy and hysterectomy for uterine leiomyosarcoma 10 years prior to presenting breast tumor. The prognostic factors associated with worse prognosis and metastasis in the uterine leiomyosarcoma are high tumor grade, age over 65, and high mitotic index.

Despite the rarity of extramammary metastasis to the breast, it is critically important to thoroughly evaluate all breast tumors to determine their origin, particularly spindle cell lesions, on the basis of the morphological features of the atypical proliferating cells; the presence of cytomorphologic atypia; tumor growth pattern, including tumor border characteristics, mitotic activity, and adjacent or admixed cells and/or tissue; and clinicoradiological features [2–4].

## 2. Case Presentation

We present the case of 51-year-old woman diagnosed with malignant lesion of the upper lateral quadrant in the right breast, 3 cm from the nipple and close to the skin. Initial ultrasonographic examination revealed a well-defined, hypoechoic, regular, ovoid, solid lesion measuring 8 mm. A subsequent mammogram showed a well-defined mass measuring 10 mm with signs of malignancy with increased vascularity (Breast Imaging Reporting and Data System category 5).

**Figure 1:**
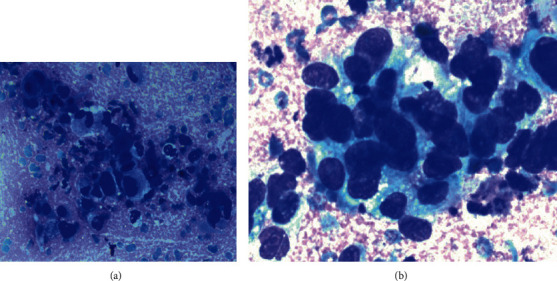
(a) Cytology shows hypercellular material containing atypical cells, irregular borders, and hyperchromasia, 10x. (b) Close view, 40x of the atypical cells with hyperchromasia and overlapping and clear nuclei.

The initial cytological analysis was obtained via fine-needle aspiration at our department, performed percutaneously using a small needle (25 gauge) and localized by ultrasound which resulted in the identification of a mesenchymal tumor with neoplastic cells with oval to spindle-shaped nuclei with moderate to high pleomorphism, irregular contours, and moderately eosinophilic cytoplasm (Figure 1). The cytology fine-needle aspiration was followed by an ultrasound-guided core biopsy.

Biopsy immunohistochemical studies indicated these cells were positive for vimentin and smooth muscle-specific markers such as desmin, caldesmon, and smooth muscle actin, but negative for cytokeratin MNF116, estrogen, progesterone receptors, and the urothelial and breast carcinoma marker GATA3. The results of the immunohistochemical studies indicate the tumor cells originated from smooth muscle, and not from epithelial breast tissue.

Finally, these cells had a Ki67 proliferation index of 20%, consistent with high levels of proliferation. The cytology report indicated a diagnosis of suspicious for malignant cell neoplasm, favoring leiomyosarcoma.

**Figure 2:**
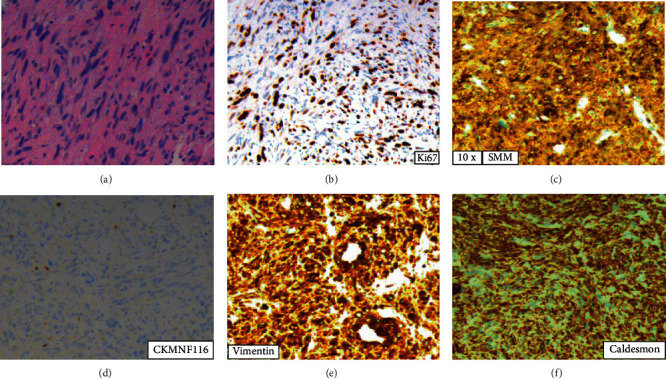
(a) Hematoxylin-eosine from the biopsy material shows solid nests of spindle cells containing fascicular growth pattern, tumor cell merge with blood vessel walls, palisading of spindle cells with eosinophilic fibrillary cytoplasm, focal granularity, and large nuclei cigar-shaped blunt-ended with variable atypia. (b) High mitotic activity is seen with Ki67. (c–f) Tumor cells were positive for smooth muscle actin, caldesmon, and vimentin indicating a nonbreast tissue origin rather than indicating a smooth muscle origin. (d) Tumor cells were negative for CKMNF116.

Histopathological examination of the tumor following lumpectomy revealed metastasis of uterine leiomyosarcoma. The resected tumor margins were clear (10 mm to the anterior plane), and the surrounding tissue was normal. Morphological findings included solid nests of spindle cells containing fascicular growth pattern, tumor cell merge with blood vessel walls, palisading of spindle cells with eosinophilic fibrillary cytoplasm, focal granularity and large nuclei cigar-shaped blunt-ended with variable atypia, cytoplasmic vacuoles at both ends of nuclei, and displaying atypical mitotic figures. High mitotic activity was seen with ~10 mitoses observed per 10 high-power fields. Central necrosis was evident and marked with fibrosis and the presence of macrophages. Tumor cells were positive for smooth muscle actin, caldesmon, calponin, and desmin which indeed indicate that the tumor is of smooth muscle origin (Figure 2).

From the histopathological perspective, in this specific case, the differential diagnosis of metastasis of uterine leiomyosarcoma included spindle cell carcinoma, malignant phyllodes tumor with stromal overgrowth, nodular fasciitis, mammary fibromatosis, myofibroblastoma, solitary fibrous tumor, primary or metastatic sarcoma, and metastatic melanoma [8, 9]. Metaplastic carcinoma was excluded based on the absence of cytokeratins MNF116 and AE1/AE3 by immunohistochemical staining. Absence of the estrogen and progesterone receptors as well as negative stain for GATA3 excluded other types of primary adenocarcinomas in the breast.

In addition, the presence of exclusively sarcomatous elements within the tumor, without epithelial structures, along with the small size of the tumor established the diagnosis of a pure sarcoma, and not a fibroepithelial one (i.e., malignant phyllodes tumor). Another important feature is that the lesion was well circumscribed and not speculated, which is more typical in primary tumors of the breast.

Although ultrasound provides meaningful information, detecting the specific features of tumors that can provide a basis for differential diagnosis presents a challenge to the radiologist but can be aided by taking a thorough review of patient history [7]. For example, the patient in this study had a history of uterine leiomyosarcoma, a disease with strong metastatic potential persisting many years after hysterectomy [10]. Further, the combination of ultrasound and mammography, as used in this study, has increased diagnostic accuracy in cases of breast cancer. These methods can be augmented with cytology, a relatively rapid and inexpensive method for providing additional diagnostic information, and that proved to be extremely useful in this case. In fact, the most useful information in the diagnosis of metastasis to the breast are the clinical history and the morphological histological features of hematoxylin and eosin- (H&E-) stained sections. If there is no history of malignancy, stain analysis may be useful in supporting origin from an extramammary site. However, it is important to consider that no marker is 100% specific or sensitive [11].

It has been also shown that metastasis could have different immunophenotype in comparison to the primary tumor. The stains to be used should be chosen based on morphology and clinical history. The recommended approach must include a panel of CK including CK7 and CK20. Breast primary cancer usually is positive for CK7 and negative for CK20. Estrogen receptor is positive in about 80% and progesterone in about 60% of mammary carcinomas. Very few tumors from other sites expressed estrogen. GATA 3 and gross cystic disease fluid protein 15 (GCDFP 15) are often expressed by carcinomas of the breast (70%), salivary glands, and skin appendages and occasionally by other carcinomas [12, 13].

## 3. Discussion

The clinical features that appear to be of importance in metastases to the breast, in general, include a rapidly growing painless, palpable, firm, round or oval, well-circumscribed, nonencapsulated nodules with a predilection for the upper outer quadrant [8–10, 14, 15], as was the case for the patient reported here. In roughly half of the reported breast metastases, tumors are adherent to the skin and superficially located, but skin changes are generally absent [15].The most common nonmammary tumors in the breast are hematological malignancies, malignant melanoma, lung tumors, renal cell carcinoma, ovarian tumors, and thyroid carcinoma [11, 15].

The more often mammographic radiological features of the metastases of the breast are well-defined or slightly irregular margins and rounded mass. The presence of calcifications is rare except for metastases from ovarian serous papillary carcinomas. Another feature is that at the time of radiological examination, usually the mass is not bilateral or multiple. Spiculation is rarely present in metastasis images in contrast with primary mammary carcinomas. Ultrasound scan typically shows a hypoechoic mass, which is sometimes heterogeneous or poorly defined appearances [11].

Past and recent studies show that a third of metastatic lesions do not show specific histological features, and clinical history is indispensable to make the correct diagnosis. However, there are specific histological features of importance in metastatic carcinoma such as the presence of clear cytoplasm, suggesting renal origin, and it is not a characteristic of primary breast carcinoma. The finding of pigment, intranuclear inclusions, and cell dissociations should include the differential diagnosis of melanoma. The poor differentiation, papillary growth features, and dedifferentiation as well psammoma bodies in a tumor are characteristics that raise the possibility of ovarian origin. Solid tumor with focal areas of ductal carcinoma in situ always will support breast origin, but it can be seen in association with metastasis from an extra mammary primary carcinoma [12, 13].

A review by Lee et al. [12] discussed about the morphological pattern of breast metastasis including the finding of a circumscribed nodule with surrounding normal breast tissue. Infiltration of cells around ducts and lobules is commonly found in lymphomas, leukaemia, and melanoma. The diagnostic problem is that it can be present in primary breast tumors as well.

The most recent World Health Organization classification of the primary mesenchymal tumors in the breast includes both leiomyosarcoma and a large variety of spindle cell lesions that represent reactive, benign, and malignant tumors with overlapping morphologic, clinicoradiological, and immunohistochemical characteristics, making a proper diagnosis potentially challenging [14].

Sarcomas in the breast include heterogeneous group of malignant tumors of mesenchymal origin, which can be primary, secondary, or metastatic from other sites. Primary sarcoma is related to the Li-Fraumeni syndrome and Neurofibromatosis 1 and environmental exposures (e.g., vinyl chloride and arsenic genetic syndromes). Secondary breast sarcoma is related to radiation or chronic lymphedema including angiosarcoma that is the most common subtype of breast sarcoma. Other common subtypes include malignant fibrous histiocytoma and fibrosarcoma [16].

Secondary tumors in the breast arising from nonmammary metastases are rare, and at the time of presentation, 90% of these patients have been diagnosed with a previous malignancy. The secondary tumor is typically detected 1-10 years following the initial cancer diagnosis. Multiple lesions in the breast are uncommon [5–7], and primary breast sarcoma is unusual, occurring in less than 1% of women with breast malignancies. Of the other spindle cell lesions of the breast, invasive carcinoma (i.e., metaplastic or sarcomatous carcinoma) is the most common and should always be considered before any other diagnosis, and it can be excluded with the use of suitable cytokeratin markers [6].

Rhabdomyosarcoma is the most commonly reported metastatic sarcoma [17], frequently in adolescents. Other sarcomas that have been described to metastasize to the breast include osteosarcoma, liposarcoma, and leiomyosarcoma. We are aware of only five cases of metastasis of uterine leiomyosarcoma to the breast [3–7, 10]. Most metastases to the breast occur in young women, likely because of a more abundant blood supply [17–20].

In most cases, sarcomas which metastasize to the breast manifest as solitary painless breast lumps, or they may also be discovered incidentally on staging imaging. Lesions may be bilateral and multiple and most commonly occur in the upper outer quadrants as describer earlier [19, 20].

Tissue diagnosis is necessary, with core biopsy establishing the diagnosis [21]. Fine-needle aspiration can be useful as in our case; however, expertise is needed to avoid false-negative rate [22]. Accurate diagnosis depends on immunohistochemistry to specify the subtype and to differentiate sarcomas from other neoplasms such as metaplastic carcinomas and phyllodes tumors, which are both positive for epithelial markers.

Metastasis to the breast from a nonbreast primary tumor typically stems from disease spread and indicates a poor prognosis; however, physician assessment of patients via both radiological and histopathological means assures a proper diagnosis, which shapes the treatment plan, thus potentially sparing the patient from unnecessary procedures such as radical mastectomy. Wide excision of the tumor resulting in cancer-free margins is recommended, when feasible. Radiation therapy and chemotherapy have also been used as adjunct therapeutic in cases of uterine leiomyosarcoma that metastasized in some cases [16–22]. Finally, we would like to stress the importance of follow-up in patients with the clinical history of this type of tumor and early detection.
